# Bispecific antibody against sclerostin and DKK1 improves bone health and reduces bone marrow adipose tissue accumulation in experimental chronic kidney disease

**DOI:** 10.1038/s41413-026-00556-y

**Published:** 2026-07-15

**Authors:** Worachet Promruk, Soher N. Jayash, Chartinun Chutoe, Hua Zhu Ke, Xiaofeng Liu, Rachel L. Wade, Alexander von Kriegsheim, William P. Cawthorn, Katherine A. Staines, Louise A. Stephen, Colin Farquharson

**Affiliations:** 1 The Roslin Institute and Royal (Dick) School of Veterinary Studies, University of Edinburgh, Edinburgh, UK; 2 Chulabhorn Royal Academy, Bangkok, Thailand; 3 Edinburgh Cancer Research UK Centre, Institute of Genetics and Cancer, University of Edinburgh, Edinburgh, UK; 4 Angitia Biopharmaceuticals (Angitia Incorporated Limited), Guangzhou, China and Westlake Village, Westlake Village, CA USA; 5 Institute for Neuroscience and Cardiovascular Research, Edinburgh Bioquarter, University of Edinburgh, Edinburgh, UK; 6 Centre for Lifelong Health, School of Applied Sciences, University of Brighton, Brighton, UK

**Keywords:** Bone quality and biomechanics, Metabolic syndrome

## Abstract

Chronic kidney disease (CKD) leads to bone loss and bone marrow adipose tissue (BMAT) accumulation. Sclerostin and dickkopf-1 (DKK1) are two inhibitors of Wnt signalling, which suppress bone formation, promote bone marrow adipogenesis, and are elevated in CKD. However, therapies targeting sclerostin have shown limited efficacy in improving bone health in CKD animal models. Herein, we explored whether dual inhibition of sclerostin and DKK1 via a rodent bispecific antibody (rbsAb) could prevent bone loss and suppress BMAT accumulation in a CKD mouse model. CKD was induced using an adenine-supplemented diet in male mice, with CKD and control mice treated weekly for 6-weeks with vehicle or 30 mg/kg body weight of rbsAb. Circulating sclerostin and DKK1 were ~2- and ~3-fold higher, respectively, in CKD mice compared to controls. Proteomic profiling by LC-MS/MS and functional enrichment analysis suggested that in CKD mice, adipogenesis, osteoclast differentiation and bone resorption were increased whereas osteoblast differentiation was inhibited. These changes were prevented by antibody treatment. MicroCT revealed that long bones of CKD mice were characterised by lower bone mineral density, trabecular and cortical bone, and impaired biomechanical properties, but their vertebrae were unaffected. RbsAb treatment prevented cortical and trabecular bone loss and restored biomechanical properties. BMAT, as visualised by microCT imaging of osmium-stained bones, was elevated in CKD but reduced to control levels by rbsAb treatment. In conclusion, dual inhibition of sclerostin and DKK1 improved bone integrity and suppressed BMAT in experimental CKD, suggesting a promising therapeutic avenue for renal osteodystrophy.

## Introduction

The progressive loss of kidney function that occurs in chronic kidney disease (CKD) leads to disturbed mineral metabolism, with CKD patients commonly presenting with hyperphosphatemia, hypercalcemia, hyperparathyroidism and increased fibroblastic growth factor-23 (FGF23) levels.^1^ These systemic changes are the primary indicators for the diagnosis of CKD–mineral bone disorder (CKD–MBD), which develops in the early stages of CKD. Disease progression results in a myriad of complications including vascular calcification and a wide spectrum of bone disorders characterised by abnormalities in bone quantity and quality, and generally referred to as renal osteodystrophy (ROD).^2,3^ The prevalence of osteoporosis in CKD patients ranges from 18% – 32% and fracture incidence of patients with end-stage kidney disease is 8-fold higher than that of the general population.^4^

A histopathological classification system based on abnormalities in bone turnover, volume and mineralisation is used to diagnose ROD and categorise it into various diseases states. These include high turnover with either fibrosis (osteitis fibrosa) or abnormal mineralisation (mixed disease), and low turnover with either normal (adynamic bone disease) or abnormal mineralisation (osteomalacia).^5^ The stimuli for increased bone remodelling are unclear, but enhanced osteoclastic bone resorption in response to elevated parathyroid hormone (PTH) is associated with a net loss of bone mass despite an accelerating bone turnover.^6–8^ Rapid remodelling also results in bone that is both immature and poorly mineralised.^3^ Nevertheless, while high-turnover bone lesions induced by secondary hyperparathyroidism are common in late-stage CKD, alternative causative mechanisms are likely as very low serum PTH levels correlate with adynamic bone disease and an increased fracture rate.^9,10^ This is clinically important, as accumulating evidence suggests that low bone turnover disease is the dominant form of ROD in the early stages of CKD, making it an ideal period to develop targeted therapeutic interventions.^10^

The factors contributing to the development of low bone turnover disease are unclear but may include elevated levels of phosphate, FGF23 and/or protein-bound uremic toxins.^11–14^ Uremic toxins can induce skeletal resistance to PTH and oxidative stress and mitochondrial dysfunction in osteoblasts/osteocytes.^15–19^ Circulating concentrations of sclerostin, a Wnt/β-catenin antagonist, are also increased in CKD patients and animal models, and these changes, which are inversely correlated to estimated glomerular filtration rate in CKD patients, are increased during adynamic bone disease and prior to rises in circulating PTH and FGF23.^20–29^ The relationship between CKD and another Wnt/β-catenin inhibitor, dickkopf-related protein 1 (DKK1), is less clear, with studies reporting unchanged,^28,30^ decreased^29,31^ or increased^26,27^ serum levels. Sclerostin binds to the Wnt co-receptor LRP 5/6 leading to the degradation of intracellular β-catenin whereas the Wnt inhibitory actions of DKK1 also involve the high-affinity transmembrane co-receptors Kremen 1/2.^32^

Disturbed Wnt/β-catenin signalling may contribute to the low bone mass characteristic of ROD. Although Sost-deficient (*Sost*^*−/−*^) mice have increased bone mass, they have limited protection against nephrectomy-induced cortical and trabecular bone loss^33,34^; however, the high bone mass of *Sost*^*−/−*^ mice at baseline may confound the interpretation of these data. Therefore, approaches using a sclerostin-neutralising antibody (Scl-Ab) such as romosozumab, which is widely recognised to increase bone mineral density (BMD) and reduce fractures in healthy men and postmenopausal women, may be more informative and offer potential therapeutic options to prevent bone loss in CKD.^35–38^

Studies on CKD patients are limited. Two studies reported that romosozumab improved BMD at the lumbar spine, total hip, and femoral neck and reduced the relative risk of new vertebral fractures in postmenopausal women with osteoporosis and mild-moderate CKD.^39,40^ However, in a pre-clinical rat model of progressive ROD, administration of a Scl-Ab provided limited protection to the architecture of the long bones and no improvement in their biomechanical properties.^26^ Interestingly, DKK1 serum levels are raised with prolonged romosozumab treatment and also in patients with sclerosteosis and van Buchem disease.^41,42^ Similarly, *Sost*^−/−^ mice and mice administered sclerostin antibody also present with a compensatory increase in the expression of DKK1^26,43,44^ raising the possibility that elevated DKK1 may attenuate the anabolic effects of sclerostin inhibition and negate major improvement in skeletal health in Scl-Ab-treated mice.^26^

In addition to bone loss, CKD can also lead to bone marrow adipose tissue (BMAT) accumulation in animal models^45–47^ and humans.^48,49^ The mechanism(s) responsible are unclear but may also involve Wnt/β-catenin signalling, which can inhibit adipogenesis and adipocyte-specific gene expression in white adipose tissue.^50–52^ Congruently, Scl-Ab treatment reduced BMAT accumulation in ovariectomised rabbits^53^ and irradiated,^54^ diabetic^55^ and rosiglitazone-treated^56^ mice; however, the changes were modest, suggesting that additional mechanisms may be involved and/or a compensatory increase in DKK1 may blunt the neutralising effects of the Scl-Ab.

The increased DKK1 expression in response to Scl-Ab treatment may limit the ability of the antibody to protect bone health in CKD mice and decrease BMAT accumulation. Therefore, in this study we tested whether a rodent bispecific antibody (rbsAb) against sclerostin and DKK1 can improve bone health and decrease BMAT accumulation in an experimental model of CKD.

## Results

### Disease profile is similar in vehicle- and rbsAb-treated CKD mice

**Fig. 1:**
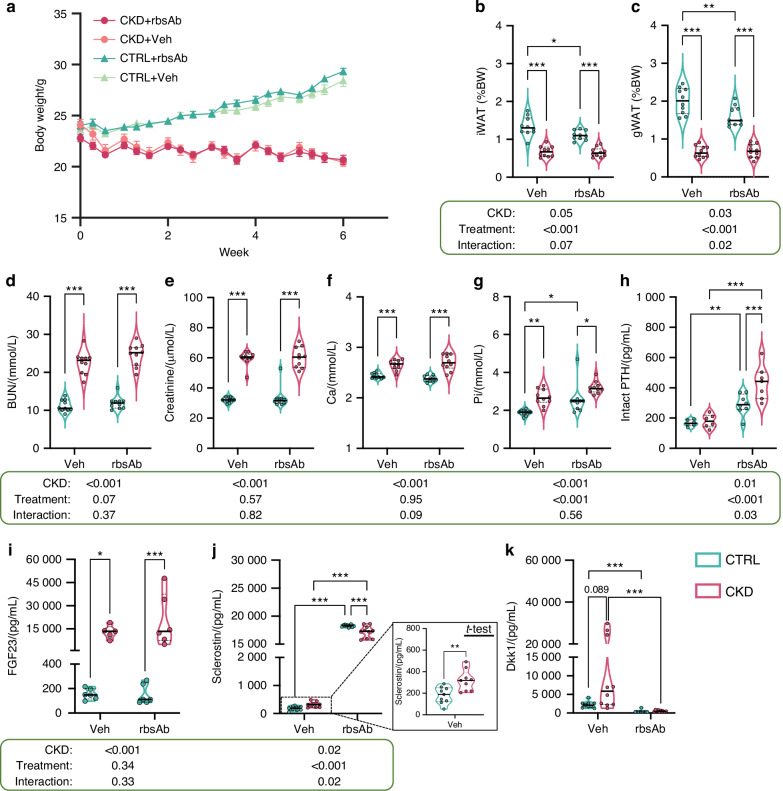
Validation of CKD development in adenine-treated mice and effect of rbsAb on plasma concentrations of sclerostin and DKK1. **a** Body weight of CKD mice was decreased over the 6-weeks of the study. The administration of antibody to either CTRL or CKD mice had no additional effect on body weight. **b**, **c** Inguinal (iWAT) and gonadal (gWAT) white adipose depots were decreased in vehicle and antibody-treated CKD mice. **d**–**g** Plasma concentrations of blood urea nitrogen (BUN), creatinine, calcium (Ca) and phosphate (Pi) were increased in vehicle and antibody-treated CKD mice. Pi levels were increased in antibody-treated control, but not CKD, mice. **h** Plasma concentrations of PTH were similar in vehicle-treated control and CKD mice but increased following antibody administration. **i** Plasma concentrations of FGF23 were increased in vehicle and antibody-treated CKD mice. **j**, **k** Plasma levels of sclerostin and DKK1 were both higher in vehicle-treated CKD compared to vehicle-treated control mice. The antibody lowered DKK1 levels in CTRL and CKD mice to levels that were indistinguishable from each other however sclerostin levels in antibody-treated mice were erroneously high, possibly due to cross-reactivity with the antigen-antibody complex and the sclerostin ELISA or alternatively a sequestration of antibody-bound sclerostin in the serum. For the data shown in the violin plots (**b**–**k**), significant effects of CKD, antibody treatment, and CKD-antibody treatment interaction were assessed using two-way ANOVA and overall *P* values for each variable, and their interactions are shown beneath each graph. For plasma DKK1 data (**k**), the Mann–Whitney test with Bonferroni correction was used to test non-normally distributed data. Significant differences between comparable groups were assessed using Tukey’s multiple comparison test and are indicated by *P < 0.05, **P < 0.01 or ***P < 0.001

We first confirmed the development of the CKD-MBD phenotype in the experimental mice. Dietary adenine-supplementation led to a loss of body weight within the first week, and by end of the study, the CKD mice were ~40% lighter than control (CTRL) mice. This was reflected in a decreased mass of the inguinal and gonadal white adipose tissue depots (iWAT and gWAT, respectively) of the vehicle-treated CKD mice (Fig. 1a–c). The CKD mice also presented with hyperphosphatemia and increased BUN, creatinine, calcium and FGF23 (Fig. 1d–g, i). Similar effects were observed between CTRL and CKD mice administered the rbsAb. The rbsAb treatment also modestly decreased iWAT and gWAT masses and increased serum phosphate in CTRL mice (Fig. 1b, c, g). Plasma PTH levels were similar in vehicle-treated CTRL and CKD mice but were raised in CKD mice treated with the rbsAb compared to similarly treated CTRL mice (Fig. 1h).

### Plasma DKK1 and sclerostin levels are elevated in CKD mice

The ability of the rbsAb to reduce circulating levels of sclerostin and DKK1 was next investigated. In vehicle-treated mice, plasma levels of sclerostin were higher in CKD compared to CTRL mice (Fig. 1j). Sclerostin concentrations in rbsAb-treated mice were extremely high, reaching supraphysiological concentrations and with no evidence of antibody-mediated neutralisation (Fig. 1j). This may be due to a sequestration of antibody-bound sclerostin in the serum, leading to elevated ELISA measurements or alternatively the antibody-sclerostin complex could cross-react with the sclerostin ELISA, as reported previously.^26,42^ In vehicle-treated mice, CKD increased median DKK1 concentrations by almost 3-fold. However, the unadjusted P value for this relatively large effect (*P* = 0.029 7) was no longer below the significance threshold after adjusting for multiple comparisons (Fig. 1k). Moreover, treatment with the rbsAb strongly and significantly decreased DKK1 concentrations in both CTRL and CKD mice to levels that were indistinguishable from each other (Fig. 1k). This demonstrates effective DKK1 neutralisation by the rbsAb. Plasma sclerostin and DKK1 levels of vehicle-treated CTRL and CKD mice were not correlated to plasma PTH levels (Fig. S1A, B).

### RbsAb treatment prevents tibial bone loss in CKD mice

**Fig. 2:**
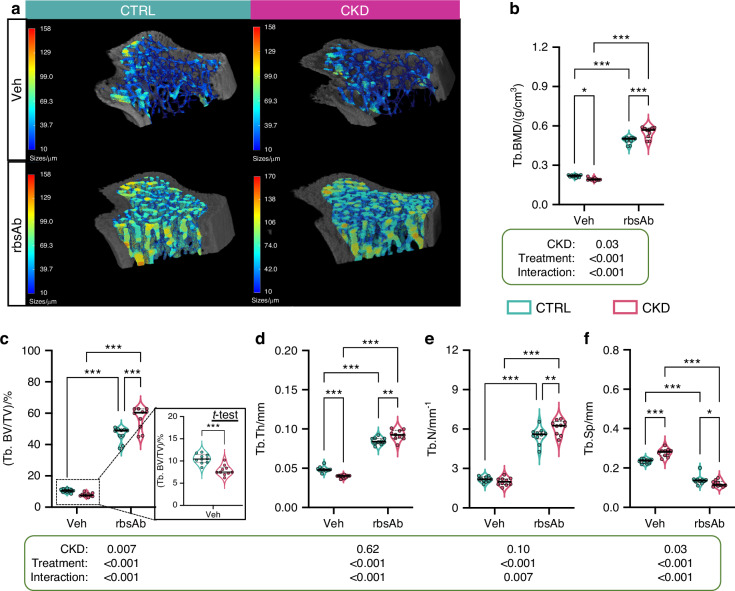
MicroCT analysis of tibial trabecular bone. **a** Representative microCT images of trabecular bone from mice of the four experimental groups. Trabecular (Tb) (**b**) bone mineral density (BMD), (**c**) bone volume fraction (BV/TV), (**d**) thickness (Th) were decreased in vehicle-treated CKD mice whereas (**F**) Tb separation (Sp) was increased in vehicle-treated CKD mice. No effect on (**e**) Tb number (N) was observed. Although antibody treatment increased Tb. BMD, BV/TV, Th, and N and decreased Tb.Sp in control and CKD mice, the effect was greater in the CKD mice. For the data shown in the violin plots (**b**–**f**), significant effects of CKD, antibody treatment, and CKD-antibody treatment interaction were assessed using two-way ANOVA and overall *P* values for each variable, and their interactions, are shown beneath each graph. Significant differences between comparable groups were assessed using Tukey’s multiple comparison test and are indicated by *P < 0.05, **P < 0.01 or ***P < 0.001

**Fig. 3:**
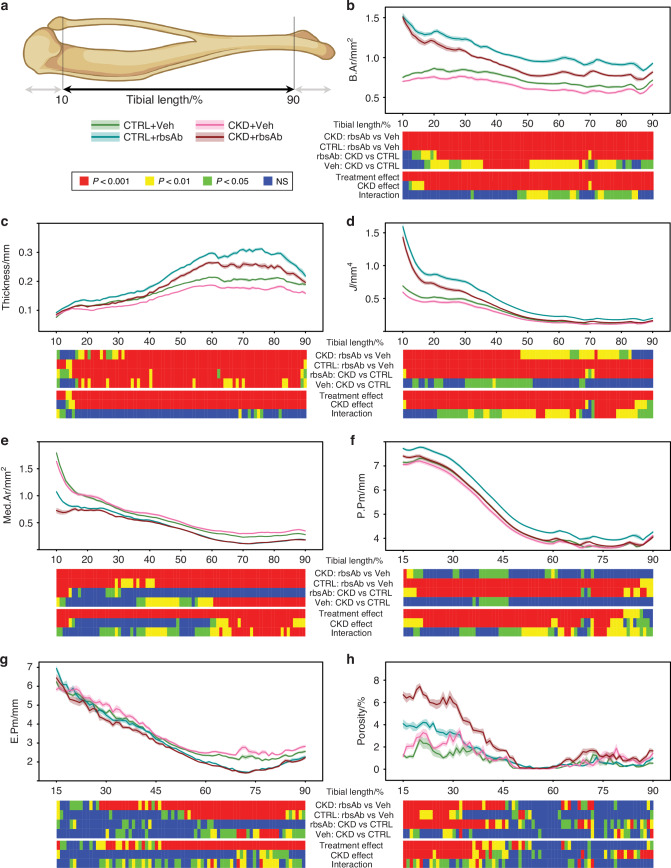
MicroCT analysis of tibial cortical bone. Quantification of whole cortical bone analysis excluding proximal and distal metaphyseal bone (**a**). Cortical (**b**) bone area (B.Ar), (**c**) thickness, (**d**) polar moment of inertia (J) and (**f**) periosteal perimeter (P.Pm) of vehicle-treated CKD mice were all reduced in various regions whereas (**e**) medullary area (Med.Ar), (**g**) endosteal perimeter (E.Pm) and (**h**) porosity were increased when compared to bones of vehicle-treated CTRL mice. Antibody treatment increased B.Ar, thickness, P.Pm, porosity and J but reduced Med.Ar and E.Pm along the tibial length in both CTRL and CKD mice

Given that CKD causes bone loss,^17,57^ we next tested if rbsAb treatment was able to prevent bone loss in the tibia and vertebrae of CKD mice. Trabecular structure and BMD of the tibia were compromised in vehicle- but not rbsAb-treated CKD mice (Fig. 2a–f). Moreover, while rbsAb administration increased trabecular BMD, bone volume fraction, thickness and number in the tibia of CTRL mice, these rbsAb effects were even greater in the CKD mice. This resulted in higher values for these skeletal properties in rbsAb-treated CKD mice compared to similarly treated CTRL mice (Fig. 2b–e). The decrease in trabecular separation in rbsAb-treated CTRL and CKD mice showed a similar effect (Fig. 2f). In cortical bone of vehicle-treated CKD mice, bone area, thickness, periosteal perimeter and polar moment of inertia were reduced, whereas medullary area, endosteal perimeter and porosity were increased when compared to the tibia of vehicle-treated CTRL mice (Fig. 3a–h). RbsAb treatment increased bone area, thickness, periosteal perimeter, porosity and polar moment of inertia but reduced medullary area and endosteal perimeter along the tibial length in both CTRL and CKD mice. The cortical bone changes in response to rbsAb treatment were similar in CTRL and CKD mice and accordingly the magnitude of differences noted between vehicle-treated CTRL and CKD mice was maintained in the rbsAb-treated mice (Fig. 3a–h). This suggests that, within long bones, CKD alters the skeletal effects of sclerostin and DKK1 in a bone-type-specific manner. Furthermore, the influence of CKD and/or rbsAb treatment on cortical architecture appeared to be site dependent. For example, differences in cortical porosity were greater in the proximal tibia (Fig. 3h), whereas more marked differences in cortical thickness were noted in the distal tibia (Fig. 3c). Neither plasma sclerostin or plasma DKK1 had any correlation with trabecular BV/TV (Fig. S1C, D).

**Fig. 4:**
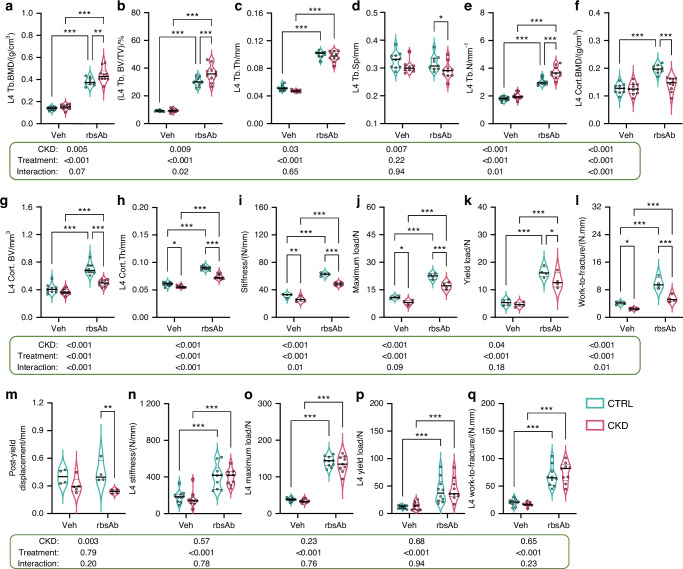
MicroCT analysis of vertebrae trabecular and cortical bone and biomechanical properties of tibia and vertebrae. Analysis of (**a**–**e**) trabecular and (**f**–**h**) cortical bone indicated that only (**h**) cortical thickness of L4 vertebrae was affected in vehicle-treated CKD mice compared to similarly treated CTRL mice. Antibody administration increased trabecular (Tb) (**a**) bone mineral density (BMD), (**b**) bone volume fraction (BV/TV), (**c**) thickness (Th), (**e**) number (**n**) and cortical (cort) (**f**) BMD, (**g**) BV and (**h**) Th in CTRL and CKD mice whereas (**d**) Tb separation (sp) was unaffected. Biomechanical analysis indicated that femur (**i**) stiffness, (**j**) maximum load, (**l**) work to fracture but not (**k**) yield load were lower in vehicle-treated CKD mice when compared to their respective CTRLs and all biomechanical properties apart from (**m**) post-yield displacement were increased in CTRL and CKD mice treated with antibody. L4 vertebrae (**n**) stiffness, (**o**) maximum load, (**p**) yield load and (**q**) work to fracture of vehicle-treated CTRL and CKD mice were similar and all biomechanical properties were increased in all mice treated with antibody. All data are shown as violin plots and significant effects of CKD, antibody treatment, and CKD-antibody treatment interaction were assessed using two-way ANOVA and overall *P* values for each variable, and their interactions, are shown beneath each graph. Significant differences between comparable groups were assess using Tukey’s multiple comparison test and are indicated by *P < 0.05, **P < 0.01 or ***P < 0.001

In L4 vertebrae, CKD had no or minimal effects on trabecular and cortical BMD and bone architecture (Fig. 4a–h). In vehicle-treated mice, only cortical thickness was decreased by CKD, whereas the effects of CKD were more pronounced in rbsAb-treated mice (Fig. 4a–h). RbsAb administration increased trabecular BMD, bone volume fraction, thickness and number whereas trabecular separation was unaffected (Fig. 4a–e). Similarly, cortical BMD, volume and thickness were also increased in response to the rbsAb (Fig. 4f–h). However, the anabolic effects of the rbsAb on vertebral trabecular and cortical bone differed between CTRL and CKD mice: for trabecular bone, the rbsAb had a stronger anabolic effect in CKD than in CTRL mice (Fig. 4a, b, d, e), while the rbsAb effects on cortical bone were stronger in CTRL than in CKD mice (Fig. 4f–h). This bone-type specific response, within the vertebrae, to rbsAb treatment is similar to that observed in the tibia (Figs. 2 and 3).

### RbsAb treatment improves tibiae and vertebrae biomechanical properties of CKD mice

As bone structure and geometry influence biomechanical properties, we next examined the response of the femur and L4 vertebrae to mechanical loading. Femur stiffness, maximum load and work to fracture were all lower in vehicle-treated CKD mice when compared to their respective CTRLs (Fig. 4i, j, l). All biomechanical properties apart from post-yield displacement were increased in rbsAb-treated CTRL and CKD mice and, in all cases, the values from the rbsAb-treated CKD mice remained lower than similarly treated CTRL mice (Fig. 4i–m) and reflect the structural changes observed in the cortical bone of the tibia (Fig. 3a–h). In contrast, compression loading of L4 vertebrae revealed that stiffness, maximum load, yield load and work to fracture were similar in vertebrae from vehicle-treated CTRL and CKD mice (Fig. 4n–q) and reflect their similar trabecular and cortical architecture (Fig. 4a–h). Similarity in biomechanical properties was also observed in vertebrae from rbsAb-administered CTRL and CKD mice, albeit all were increased when compared to their respective vehicle-treated mice (Fig. 4n–q).

### Gene and proteomic profiling of cortical bone reveals rbsAb-mediated prevention of CKD-induced dysregulation of osteoblast differentiation

**Fig. 5:**
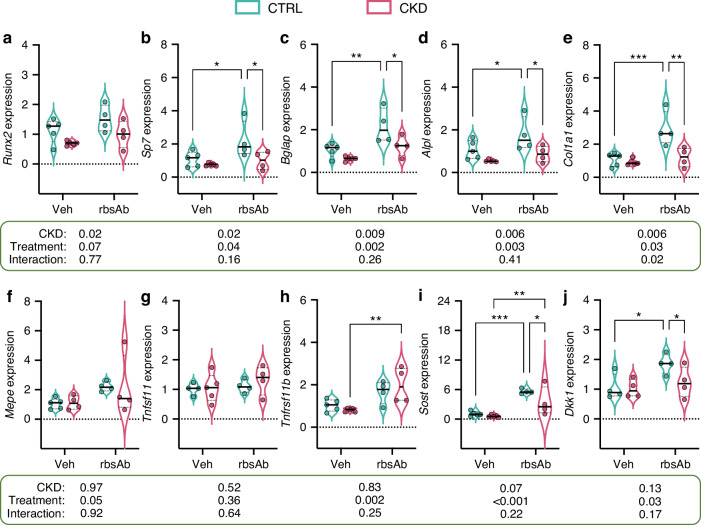
Osteogenic gene expression in cortical bone of the tibia. The expression of the osteoblast/osteocyte genes (**a**) *Runx2*, (**b**) *Sp7*, (**c**) *Bglap* (**d**) *Alpl* (**e**) *Col1a1* (**f**) *Mepe* (**g**) *Tnfsf11* (**h**) *Tnfrsf11b* (**i**) *Sost* and (**j**) *Dkk1* were similar in vehicle-treated CTRL and CKD mice. Antibody treatment increased the expression of *Sp7, Bglap, Alpl, Col1a1* and *Dkk1* in CTRL mice, *Tnfrsf11b* expression in CKD mice and *Sost* expression CTRL and CKD mice. *Runx2, Mepe* and *Tnfsf11* expression by CTRL and CKD mice were altered by antibody treatment. All data are shown as violin plots and significant effects of CKD, antibody treatment, and CKD-antibody treatment interaction were assessed using two-way ANOVA and overall *p* values for each variable, and their interactions, are shown beneath each graph. Significant differences between comparable groups were assess using Tukey’s multiple comparison test and are indicated by *P < 0.05, **P < 0.01 or ***P < 0.001

To further understand the cellular events responsible for bone loss in CKD mice and its prevention by rbsAb administration, we performed gene expression and proteomic analyses of cortical bone. The expression of osteoblast/osteocyte genes was similar in tibial cortical bone of vehicle-treated CTRL and CKD mice (Fig. 5a–j), while the effects of rbsAb treatment were variable: it increased the expression of *Sost* in both CTRL and CKD mice (Fig. 5I); increased *Sp7, Bglap, Alpl, Col1a1* and *Dkk1* in tibiae of CTRL mice but not CKD mice (Fig. 5b–e, j); and increased *Tnfrsf11b* expression in CKD mice only (Fig. 5h).

**Fig. 6:**
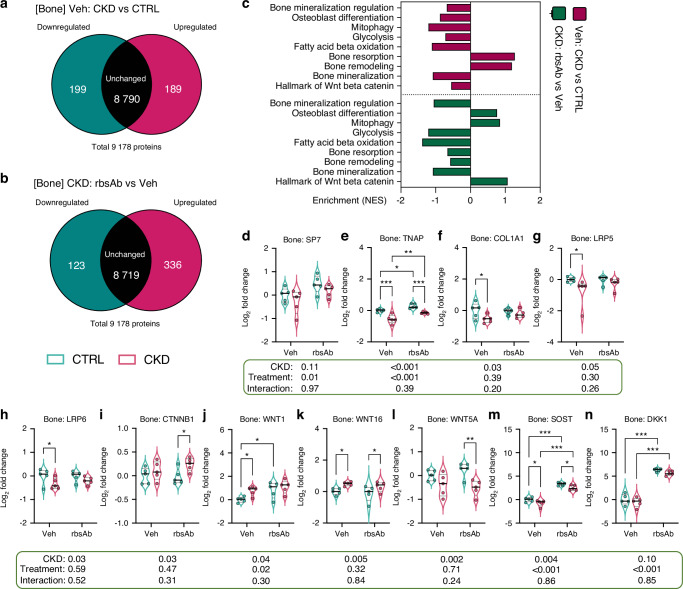
Proteomic profiling of cortical bone. **a**, **b** Venn diagrams illustrate global differential protein expression in the tibia of CKD mice and mice treated with antibody. **c** Gene set enrichment analysis identified the osteoblast/osteocyte-related pathways implicated in the aetiology of ROD and those affected by antibody treatment. From the proteomics analysis the expression of some of the significantly altered proteins associated with osteoblast differentiation such as (**e**) TNAP and (**f**) COL1A1, but not (**d**) SP7 were reduced in vehicle-treated CKD mice. The expression of proteins involved in Wnt-signalling, (**g**) LRP5, (**h**) LRP6, and (**m**) SOST were reduced in vehicle-treated CKD mice whereas (**j**) WNT1 and (**k**) WNT16 were increased and (**i**) CTNNB1, (**l**) WNT5a and (**n**) DKK1 were unchanged. Antibody treatment increased (**e**) TNAP, (**m**) SOST and (**n**) DKK1 expression in both control and CKD bones. Comparison of the expression of individual proteins are shown as violin plots and significant effects of CKD, antibody treatment, and CKD-antibody treatment interaction were assessed using two-way ANOVA and overall *P* values for each variable, and their interactions, are shown beneath each graph. Significant differences between comparable groups were assessed using Tukey’s multiple comparison test and are indicated by *P < 0.05, **P < 0.01 or ***P < 0.001

The proteomic analysis by LC-MS/MS revealed that 199 proteins were down regulated and 189 proteins were upregulated in bone from vehicle-treated CKD mice compared to vehicle-treated CTRL mice (Fig. 6a and Fig. S2A). Moreover, in CKD mice, the expression of 123 proteins was downregulated and 336 proteins were upregulated in bone by the administration of rbsAb compared with vehicle (Fig. 6b and Fig. S2B). To identify the osteoblast/osteocyte-related pathways implicated in the aetiology of ROD, we performed functional enrichment analysis. From the top ontology terms, the osteoblast differentiation and hallmark of Wnt/β-catenin pathways were depleted, whereas both bone resorption and bone remodelling pathways were enriched in vehicle-treated CKD mice (Fig. 6c). The enrichment analysis of CKD mice further indicated that the rbsAb was able to prevent the defective osteoblast differentiation, Wnt/β-catenin activity and excessive bone resorption and bone remodelling which are consistent with the increased cortical bone mass observed in the rbsAb-treated mice (Fig. 3). Given that the osteoblast differentiation pathway was notably affected by CKD and rbsAb treatment, we further assessed the expression patterns of some of the significantly altered proteins associated with this pathway (Fig. 6d–n). The expression of COL1A1 and TNAP was both downregulated in vehicle-treated CKD mice, but the osteoblast transcription factor, SP7, was unchanged (Fig. 6d–f). With regard to Wnt signalling, the expression of LRP5, LRP6 and SOST was decreased, WNT1 and WNT16 increased, and CTNNB1, WNT5A and DKK1 were unchanged in vehicle-treated CKD mice (Fig. 6g–n). The rbsAb increased TNAP, SOST and DKK1 expression, which is in accord with the gene expression results (Fig. 5d, i, j).

### RbsAb treatment prevents BMAT accumulation in CKD mice

**Fig. 7:**
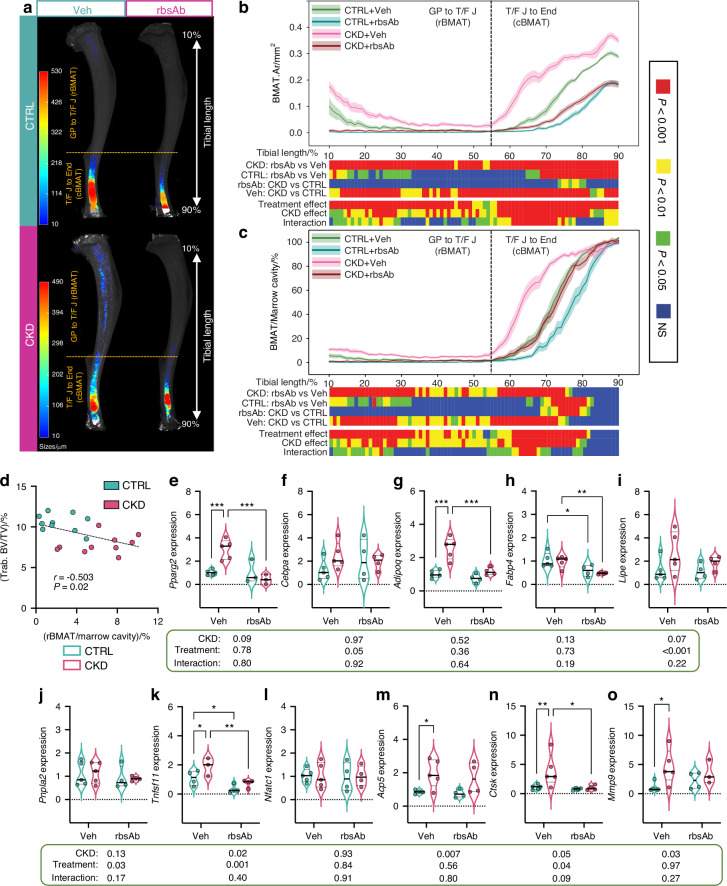
BMAT distribution along the tibial bone shaft and adipogenic and osteoclastogenic gene expression in bone marrow tissue. **a** Visualisation of osmium-stained bone marrow adipose tissue (BMAT) present between the growth plate to tibia/fibula junction (GP-T/F J), which contains regulated BMAT (rBMAT) and the tibia/fibula junction to the end of distal bone (T/F J-End), which contains constitutive BMAT (cBMAT) of vehicle and antibody treated CTRL and CKD mice. **b** Total BMAT and (**c**) BMAT normalised to bone marrow area (with trabecular bone excluded) was increased in vehicle-treated CKD mice compared to similarly treated CTRL mice. Antibody treatment reduced BMAT accumulation along the entire length of the tibia of CTRL and CKD mice. **d** Spearman correlations between trabecular bone volume fraction (Tb.BV/TV) and rBMAT indicated a negative correlation. The expression of the adipogenic genes (**e**) *Pparg2* and (**g**) *Adipoq* but not (**f**) *Cebpa*, (**h**) *Fabp4*, (**i**) *Lipe* or (**j**) *Pnpla2* was increased in vehicle-treated CKD mice. Antibody treatment had no effect on the expression of *Cebpa, Lipe* or *Pnpla2* but the difference in expression of *Pparg2* and *Adipoq* seen in the vehicle-treated mice was abolished by antibody treatment. The expression of osteoclast genes (**k**) *Tnfsf11*, (**m**) *Acp5*, (**n**) *Ctsk*, and (**o**) *Mmp9* was increased in vehicle-treated CKD mice, and this difference in expression was nullified by antibody treatment. **l** *Nfatc1*, was similar in CTRL and CKD samples and was not affected by antibody treatment. For the data shown in the violin plots (**e**–**o**) the significant effects of CKD, antibody treatment, and CKD-antibody treatment interaction were assessed using two-way ANOVA and overall *P* values for each variable, and their interactions, are shown beneath each graph. Significant differences between comparable groups were assessed using Tukey’s multiple comparison test and are indicated by *P < 0.05, **P < 0.01 or ***P < 0.001

We next quantified BMAT along the tibial length as visualised by microCT imaging of osmium-stained bones to determine if the accumulation of BMAT that occurs in CKD animal models and patients was prevented by rbsAb treatment.^45,47,49^ Total BMAT area, or BMAT area normalised to bone marrow area, of vehicle-treated CKD mice was increased compared to vehicle-treated CTRL mice (Fig. 7a–c). RbsAb treatment reduced BMAT accumulation along the entire length of both CTRL and CKD tibiae (Fig. 7a–c). Moreover, rbsAb treatment of CKD mice decreased the relative BMAT volume to levels similar to those observed in vehicle-treated CTRL mice (Fig. 7c). Correlation analysis revealed an inverse relationship between rBMAT and trabecular BV/TV, which is in agreement with previous studies^47^ (Fig. 7d). Bone marrow adiposity can be decreased by PTH,^58,59^ but no correlation was found between plasma PTH and rBMAT (Fig. S1E); this is in broad agreement with prior observations.^48,60^ Similarly, neither plasma sclerostin nor plasma DKK1 had any correlation with rBMAT (Fig. S1F, G).

### Bone marrow adipocyte and osteoclast gene and protein expression are dysregulated in CKD but normalised by rbsAb treatment

We next investigated the cellular and molecular mechanisms responsible for the accumulation of BMAT in CKD and its prevention by the rbsAb. To do so, we assessed the expression of key adipocyte transcription factors and phenotype-specific genes and proteins in bone marrow from CTRL and CKD mice, both with and without rbsAb treatment. In comparison to vehicle-treated CTRL mice, the expression of *Pparg2* and *Adipoq*, but not *Cebpa*, *Fabp4* or the lipase-encoding genes, *Pnpla2* and *Lipe*, was increased in vehicle-treated CKD mice (Fig. 7e–j). RbsAb treatment downregulated *Pparg2* and *Adipoq* expression in CKD mice, resulting in similar levels to those in rbsAb -treated CTRL mice; this is consistent with the effects on relative marrow adiposity (Fig. 7c). *Fabp4* expression was also downregulated by antibody treatment of both CTRL and CKD mice (Fig. 7h). Sclerostin can increase the RANKL/OPG ratio in bone marrow stromal cells and promote their differentiation into osteoclasts; thus, we also quantified the expression of osteoclast transcription factors and mature osteoclast genes.^61,62^ The expression of the master transcription regulator of osteoclast differentiation, *Nfatc1*, was similar in CTRL and CKD samples and was not affected by rbsAb treatment (Fig. 7l). In contrast, the expression of *Ctsk, Mmp9, Acp5* and *Tnfsf11* was increased by CKD in vehicle-treated mice and rbsAb treatment reduced Tnfsf11 and Ctsk expression in CKD samples resulting in similar expression levels between CTRL and CKD mice (Fig. 7k–o).

**Fig. 8:**
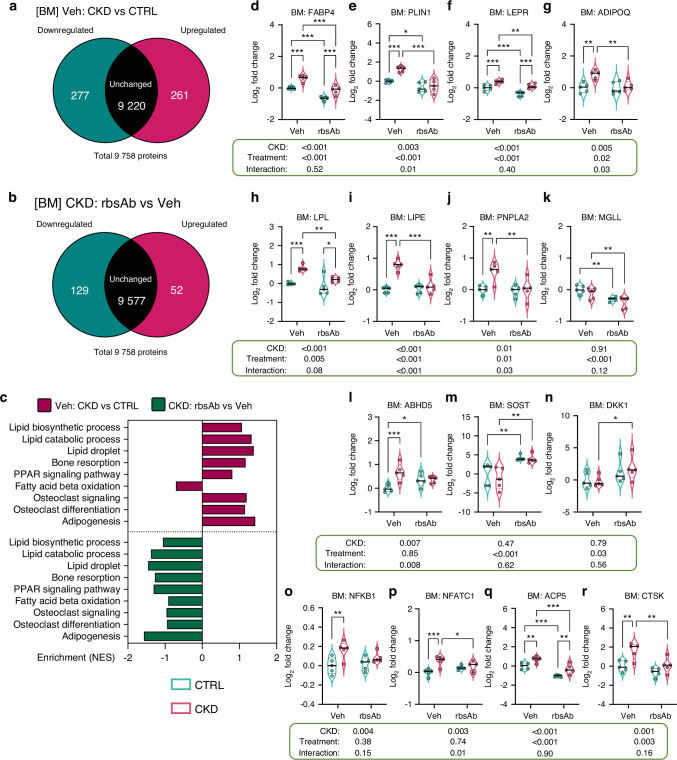
Proteomic profiling of bone marrow. **a**, **b** Venn diagrams illustrate global differential protein expression in the bone marrow of CKD mice and mice treated with antibody. **c** Gene set enrichment analysis revealed that gene sets associated with adipogenesis, lipid metabolism, osteoclast differentiation and signalling, and bone resorption pathways were enriched in vehicle-treated CKD mice as compared to similarly treated CTRL mice. Antibody treatment reversed these pathway changes in CKD mice. The expression of proteins associated with adipocyte differentiation and lipid metabolism including (**d**) FABP4, (**e**) PLIN1, (**f**) LEPR, (**g**) ADIPOQ and (**h**) LPL (**i**) LIPE, (**j**) PNPLA2, (**k**) MGLL and (**l**) ABHD5, was increased in vehicle-treated CKD mice compared to vehicle-treated control mice. This increased expression was decreased by antibody treatment. In addition, CKD increased the expression of (**o**) NFKB1, (**p**) NFATC1, (**q**) ACP5 and (**r**) CTSK, which are critical for osteoclast differentiation and bone resorption, while antibody treatment normalized (**o**) NFKB, (**p**) NFATC and (**r**) CTSK but not (**q**) ACP5. **m**, **n** SOST and DKK1 expression was similar in vehicle-treated CTRL and CKD mice and increased with antibody treatment. Comparison of the expression of individual proteins is shown as violin plots and significant effects of CKD, antibody treatment, and CKD-antibody treatment interaction were assessed using two-way ANOVA and overall *P* values for each variable and their interactions are shown beneath each graph. Significant differences between comparable groups were assessed using Tukey’s multiple comparison test and are indicated by *P < 0.05, **P < 0.01 or ***P < 0.001

We further interrogated these bone marrow effects using proteomics. The volcano plots (Fig. S2C, D) and Venn diagrams (Fig. 8a, b) illustrate the global proteomic alterations observed under CKD conditions and the modulatory effects of rbsAb treatment in CKD mice. Functional enrichment analysis of the top ontology terms disclosed that, under vehicle treatment, adipogenesis and lipid metabolism, osteoclast differentiation and signalling, and bone resorption pathways were enriched in CKD vs CTRL mice (Fig. 8c). These enriched pathways were depleted by rbsAb treatment of CKD mice (Fig. 8c) and together these data are in accord with the ability of the rbsAb to protect CKD mice from bone loss and BMAT accumulation (Figs. 2, 3 and 7). Adipocyte proteins were then analysed, which illustrated upregulation of FABP4, PLIN1, LEPR, ADIPOQ, LPL, LIPE, PNPLA2 and ABHD5 in CKD mice. These increases were significantly prevented by rbsAb treatment, thereby cancelling or minimising the difference in their expression levels between CTRL and CKD mice (Fig. 8d–l). In addition, proteins associated with osteoclast differentiation and bone resorption were upregulated in vehicle-treated CKD vs CTRL mice (Fig. 8o–r). This CKD effect was generally prevented by rbsAb administration, resulting in expression levels of NFKB, NFATC and CTSK that were similar between rbsAb -treated CTRL and CKD mice (Fig. 8o–r). SOST and DKK1 expression were similar in vehicle-treated CTRL and CKD mice, but, in agreement with the bone gene and protein expression data (Figs. 5i, j and 6m, n), the expression of both β-catenin inhibitors was increased by rbsAb treatment (Fig. 8m, n).

## Discussion

Therapies for the treatment of ROD are currently focussed on the use of calcitriol and calcimimetics to lower circulating levels of PTH and reduce the adverse skeletal effects of secondary hyperparathyroidism.^63,64^ This approach is recommended prior to consideration of other more targeted interventions, but as elevated PTH levels are a poor indicator of low bone turnover disease, there is a requirement for alternative treatments that can improve bone health regardless of PTH status.^9^ Furthermore, CKD often coexists with osteoporosis in the ageing population, resulting in an increased fracture risk in patients with these combined comorbidities.^65^ While there are some recognised limitations and caveats, many established therapeutic approaches to osteoporosis can improve bone health in CKD patients.^66^ In patients with mild to moderate CKD, bisphosphonates raise BMD and reduce fractures, but their administration to patients with pre-existing very low bone turnover is a concern, as is their accumulation in CKD patients with impaired renal clearance.^67,68^ Other anti-osteoporosis drugs such as denosumab, teriparatide and abaloparatide increase BMD and reduce fracture risk in patients with mild to moderate CKD. However, PTH analogues may aggravate existing hyperparathyroidism and denosumab, like bisphosphonates, can induce hypocalcemia.^66,69–71^

An attractive alternative approach may involve the neutralisation of sclerostin, an inhibitor of bone formation and recognised to be elevated in bone^3,20^ and serum^20,22,25^ of CKD patients. Romosozumab is a humanised antibody that targets sclerostin, resulting in the transient activation of bone formation and inhibition of bone resorption.^35,72^ Romosozumab can reduce fracture risk in CKD, but evidence is limited to a recent post hoc analysis of patients from the phase 3 clinical trials, FRAME and ARCH.^39^ Preclinical studies have also reported that romosozumab can improve bone health in murine models of diabetes and CKD.^73,74^ A limitation of targeting sclerostin alone to promote bone formation is the upregulation of DKK1 expression, which may dampen the bone formation response to sclerostin neutralisation.^26,42–44^ Therefore, one of the aims of this present study was to examine the ability of a bispecific antibody to sclerostin and DKK1 to improve bone health in a mouse model of CKD.

It has been widely reported that the structure and biomechanical properties of long bones are compromised in CKD rodents.^17,75–77^ A role for secondary hyperparathyroidism is often implicated in the progression of ROD, but structural and mechanical integrity defects are also found in the tibia of this study, despite normal levels of circulating PTH. However, in contrast to previous animal studies in which PTH levels were elevated, we do not observe structural or biomechanical abnormalities in the vertebrae of the CKD mice.^78–80^ The gene expression and functional pathway analysis suggest that rbsAb treatment promotes osteoblast differentiation and inhibits bone resorption, and this is consistent with the rbsAb profound effects on both cortical geometry and trabecular architecture of the vertebrae and long bones of CTRL and CKD mice. The efficacy of the rbsAb to improve biomechanical and structural properties of the tibia of CKD mice is in stark contrast to that observed in a Cy/+ rat model of CKD where the effects of a Scl-Ab on the structure and biomechanical properties of vertebrae and long bones were limited and only observed in low PTH conditions.^26,78^ Although the different rodent models used in this study and that of Moe and colleagues^26^ may explain their altered osteoanabolic response to rbsAb or Scl-Ab, both the adenine and Cy/+ models present with progressive biochemical abnormalities and bone loss that parallel advanced CKD in humans. The ability of the rbsAb to produce a robust osteoanabolic effect in a CKD model is a novel observation and is consistent with previous studies where a bispecific antibody targeting sclerostin and DKK1 was more effective than monotherapy treatment in increasing BMD, biomechanical properties and bone repair activity in rodents and nonhuman primates.^43,81–83^ Also, a 3:1 mixture of Scl-Ab and DKK1-Ab promotes the formation of 2–3 times more trabecular bone than an equivalent dose of Scl-Ab alone.^84^ These observation are interesting as DKK1-Ab on its own has no consistent osteoanabolic effects in rodents although in small clinical trials the DKK1 monoclonal antibody, BHQ880, alone or in combination with zoledronic acid, increased anabolic bone activity in patients with relapsed/refractory or smouldering multiple myeloma.^43,84–88^ The increased cortical porosity in the rbsAb-treated mice was however, unexpected, as previous studies have indicated that when corrected for bone volume, cortical porosity in *Sost*^*−/−*^ mice was similar to wild-type mice.^89^

In this study, we also aimed to verify if the rbsAb was able to reduce the amount of BMAT that is known to accumulate in CKD patients and animal models.^45,46,48,49^ Though Wnt/β-catenin signalling is known to block expression of C/EBPα and PPARγ in white adipocytes and the loss of Wnt/β-catenin signalling causes a cell fate shift of pre-osteoblasts from osteoblasts to adipocytes, less is known about the regulatory effects of this pathway on bone marrow adipocytes (BMad).^90,91^ Sclerostin levels positively associate with higher vertebral marrow fat in men and in cell culture studies, adipogenesis is promoted in sclerostin challenged bone marrow mesenchymal stromal cells.^92,93^ Decreased BMAT accumulation in *Sost*^*−/−*^ mice provides further evidence that BMad accumulation in CKD could be a consequence of increased sclerostin levels.^92^ Nevertheless, romosozumab has no effect on bone marrow adiposity of the iliac crest in postmenopausal osteoporotic women, and the ability of Scl-Ab to decrease BMAT in irradiated or rosiglitazone-treated mice was modest and limited to specific regions of the long bones.^54,56,94^ These subtle, depot-specific effects of Scl-Ab on BMad contrast with the results of the present study. Gene and protein expression data suggest an inhibition of bone marrow adipogenesis with rbsAb treatment and this is consistent with the reduced BMAT accumulation along the entire tibia of rbsAb -treated CTRL and CKD mice. The decrease in total BMAT in rbsAb -treated mice is similar to that reported in Scl-Ab-treated normal adult rats^95^ but this reduction may in part be a consequence of an increase in trabecular bone volume and a corresponding reduction in marrow space in which adipocytes can inhabit. However, when normalised to marrow area, BMAT is still decreased in rbsAb -treated mice and suggests that an increase in Wnt/β-catenin signalling has a direct negative effect on BMad, independent of bone changes. When normalised to marrow area a reduction in BMAT was not observed in Scl-Ab-treated rats^95^ and suggests that the ability of the Scl-Ab alone to restore normal amounts of BMAT is impeded by a compensatory increase in the expression of DKK1.

Wnt-pathway inhibition increases gradually as kidney function declines and is considered an early event in the pathogenesis of CKD.^24^ Nevertheless, the relationship between blood sclerostin and PTH levels in CKD patients and animal models is unclear. A positive, negative or, as found in this present study, no correlation between circulating levels of sclerostin and PTH have been reported.^26,30^ Although PTH is known to supress *Sost* and *Dkk1* expression by bone cells,^96,97^ a regulatory role for PTH in this study is unclear as circulating PTH levels in the CKD mice is normal. Possibly, the chronic elevation of circulating sclerostin and DKK1 in CKD overwhelms the ability of PTH to regulate their circulating levels leading to PTH resistance and the promotion/aggravation of adynamic bone disease as well as an inability to decrease marrow adipogenesis.^58,59,98^

Nevertheless, an explanation for the higher circulating levels of sclerostin and DKK1 in CKD remains unclear.^20–29^ Reduced renal excretion is unlikely,^25^ and others have reported immunohistochemistry data indicating increased osteocyte expression of sclerostin in CKD.^3,20,25^ Alternatively, the increased expression of sclerostin and DKK1 may involve TGF-β, which is elevated in serum and bone in CKD and promotes osteocyte sclerostin expression.^99,100^ Furthermore, sclerostin levels are positively correlated with inflammation markers, phosphate and uremic toxins, whereas FGF23 can induce DKK1 and inhibit osteoblast Wnt/β-catenin signalling via a soluble Klotho/MAPK–mediated process.^97,101,102^ The upregulation of *Sost* and *Dkk1* expression and their encoded proteins in bone of rbsAb-treated mice may be a consequence of lowered circulating levels of the proteins, although for sclerostin, we were unable to confirm this due to possible sequestration of antibody-bound sclerostin in the serum or ELISA cross-reactivity with the bispecific antibody.^26,42^ A similar increase in osteocyte *Sost* and *Dkk1* expression has been observed previously in Scl-Ab-treated CKD rats and mice treated with antibodies targeting both sclerostin and DKK1.^26,43,84^ Also, in vehicle-treated CKD mice, the rise in circulating levels of sclerostin and DKK1 did not correspond to expression levels in cortical bone. These observations are in agreement with the notion of self-regulation of bone formation previously proposed for Wnt inhibitors.^103^

While the rbsAb improves bone health and decreases BMad accumulation in an experimental model of CKD, it is important to recognise that there may be limitations to this possible therapeutic approach. Cardiovascular adverse events have been reported with romosozumab in some (ARCH and BRIDGE) but not all (FRAME) clinical trials.^36–38,104^ The presence of sclerostin^105,106^ and DKK1^107^ in cardiovascular tissue may protect against vascular calcification and while theoretically, romosozumab inhibition may aggravate the progression of vascular calcification in CKD, there is no evidence of aortic mineralisation in rats or cynomolgus monkeys treated long-term with romosozumab.^108–110^ Furthermore, neutralisation of DKK1 prevents vascular calcification in mice with renal insufficiency^27^ and to the best of our knowledge, cardiovascular events have not been reported in *Sost*-deficient mice or individuals with sclerosteosis or van Buchem disease.^111–113^ Nevertheless, further animal and clinical studies focused on potential vascular effects in the setting of sclerostin and/or DKK1 blockade are required to evaluate the effect of prolonged treatment on cardiovascular health.

There are some limitations to this study. The inclusion of additional groups of mice treated with agents having mono-specificity against either sclerostin or DKK1 would have allowed us to make direct comparisons on the efficacy of the different treatments in the same CKD model. Also, as women have a higher prevalence of CKD than men, the inclusion of male and female mice would have allowed us to explore the effectiveness of the rbsAb in preventing bone loss and BMAT accumulation in both genders.^114^ The study of male and female mice would also have allowed us to examine the reported sex-specific differences in the skeletal responses to Wnt-pathway therapies.^115^

In conclusion, mice fed an adenine-enriched diet present with BMAT accumulation, trabecular and cortical bone loss and impaired biomechanical properties. A bispecific antibody to sclerostin and DKK1 was able to improve bone structure and biomechanical properties of bone and suppress BMAT accumulation. These results highlight a novel therapeutic strategy to enhance bone health in patients with CKD and pave the way for future translational applications in ROD management.

## Materials and methods

### Mice

To induce CKD, eight-week-old male C57BL/6JCrl mice (Charles River Laboratories, Margate, UK) were fed a diet supplemented with 0.2% adenine for 6-weeks (Envigo, Bicester, UK). Each week the adenine diet was offered for 5-days and replaced by a normal diet for 2 days. This modification of our previous protocol was based on the studies of Lair and colleagues and was introduced to induce CKD but avoid pathological weight loss.^57,116^ The CTRL mice received the same diet without adenine (Envigo). The bispecific antibody against sclerostin and DKK1 (Angitia Biopharmaceuticals, Guangzhou, China) or vehicle (PBS) was administered (30 mg/kg body weight) by subcutaneous injection to CTRL and CKD mice once a week for 6 weeks (*n* = 10/group). Body weights were obtained from mice twice weekly until sacrifice at 14-weeks of age. All mouse studies were approved by the University of Edinburgh Animal Welfare and Ethical Review Board and were conducted under a project license granted by the UK Home Office. Animal studies were conducted and are reported in line with the ARRIVE guidelines.

### Plasma biochemistry

All mice were sacrificed after 6-weeks treatment and blood was collected by cardiac puncture under terminal anaesthesia. Plasma creatinine, blood urea nitrogen (BUN), phosphate and calcium were quantified using a biochemistry analyser (Beckman Coulter AU480). Intact PTH (QuidelOrtho, San Diego, USA), FGF23 (Kainos Laboratories, Inc. Japan), sclerostin and DKK1 (R&D Systems, Abbington, UK) were measured by ELISA according to the manufacturers’ instructions.

### Micro computed tomography (microCT)

The changes in trabecular and cortical bone structure and BMD of L4 vertebrae (unfixed) and left tibia (fixed in 10% formaldehyde for 24 h) were assessed by microCT (NeoScan N80, Mechelen, Belgium). Briefly, the bones were scanned with an isotropic voxel size of 5 μm (60 kV, 167 μA and 0.5 mm aluminium filter, 0.6° rotation angle) and the scans were reconstructed using the NRecon 1.7.3.0 programme (Bruker, Kontich, Belgium) to remove artefacts, including beam-hardening and ring artefacts. CTAn software 1.15.4.0 (Skyscan, Kontich, Belgium) was used to evaluate bone histomorphometric parameters. To generate three-dimensional (3D) images, the scans were reconstructed and 3D images were created using the Neoscan80 software package (NeoScan).

For L4 vertebrae, a 300-slice subset through the middle of the vertebrae’s body was analysed and the following trabecular [bone mineral density (BMD; g/cm^2^), bone volume/tissue volume (BV/TV; %), thickness (Tb. Th; mm), number (Tb. N; 1/mm) and separation (Tb. Sp; mm)] and cortical [BMD; g/cm^2^, bone volume (BV; mm^3^) and Th (mm)] parameters were calculated.^56^ Each tibia was aligned along its longitudinal axis, and the trabecular volume of interest (VOI) in the proximal metaphysis was a 1 000-µm section of the metaphysis, 250 µm subjacent to the growth plate. The same trabecular parameters measured in the L4 vertebrae were quantified in the tibial reconstructions. For cortical analysis of the tibia, the proximal and distal portions were digitally cropped to exclude the epiphysis, growth plates and trabecular bone from the analysis.^57^ Bone area (B.Ar; mm^2^), thickness (Th; mm), polar moment of inertia (J; mm^4^), medullary area (Med.Ar; mm^2^), periosteal perimeter (P.Pm; mm), endosteal perimeter (E.Pm; mm) and porosity (%) were determined. Hydroxyapatite phantoms of known densities (0.25 and 0.75 g/cm^3^) were scanned and reconstructed under identical conditions as the experimental samples to allow the calculation of BMD. R studio was used to create the line graph of the cortical bone parameters along the tibia length.^55,58^

### Quantification of BMAT

After initial microCT scanning, the bone marrow adipocytes within the left tibiae were stained with osmium tetroxide as previously described.^47^ In brief, the decalcified bones were incubated with 1% osmium tetroxide for 48 h, washed and stored in Sorensens’ buffer at 4 °C. The osmium-stained bones were re-scanned by microCT, and total BMAT area was calculated as well as being normalised to the size of the bone marrow cavity, with trabecular bone excluded. BMAT volume was quantified in two distinct anatomical regions: the growth plate to tibia/fibula junction (GP-T/F J), which contains regulated BMAT (rBMAT); and the tibia/fibula junction to the end of distal bone (T/F J-End), which contains constitutive BMAT (cBMAT).^117^ R studio was used to create the line graph of BMAT accumulation along the tibia length.

### Biomechanical testing of tibia and vertebrae

The L4 vertebrae and right femora were stored, unfixed, at −20 °C in water and their biomechanical properties were evaluated by a LS5 Lloyds materials testing machine with NEXYGEN Plus software (Ametek, Leicester, UK). For the 3-point bending, the femora were positioned horizontally on custom supports and a 100 N load cell was applied perpendicular to the mid-diaphysis at a speed of 10 mm/min. For compression loading, the vertebral body was isolated from the spinal processes and prepared with flat and parallel ends using a polishing wheel and finally bonded to a fixed bottom plate with cyanoacrylate glue. A 500 N load cell at a speed of 10 mm/min compressed the vertebra. Each femur and vertebra were tested to fracture, and data were recorded after every 0.2 N change in load. The load–displacement curve for each bone was analysed, and stiffness, maximum load, yield load, work to fracture and post-yield displacement were calculated.^118^

### RT-qPCR

The proximal and distal ends of the right tibiae were removed, and the bone marrow was flushed out by centrifugation. Both the tibial shaft and the bone marrow were snap frozen in liquid nitrogen and stored at −80 °C. The tissue was homogenised by a Rotor-Stator Homogenizer and RNA was extracted using a Qiagen RNeasy Mini kit (Qiagen, Manchester, UK) and quantified by nanodrop spectrophotometry (Thermo Fisher Scientific, Loughborough, UK). RNA quality was evaluated by the 260/280 nm ratio. After reverse transcription, gene expression was quantified using the SYBER green method and an Agilent Aria 2.1 real-time qPCR system (Agilent Technologies, Cheadle, UK). Target gene expression was normalised to a housekeeping gene (*Ppia*) and analysed using the 2^−ΔΔCt^ method. Oligonucleotide primers (Table S1) were obtained from Sigma-Aldrich (Gillingham, UK) and Thermo Fisher Scientific.

### Proteomics

Both proximal and distal ends of the right tibia were removed, and bone morrow isolated by centrifugation. Cortical bone and bone marrow tissue were homogenised by a Rotor-Stator Homogenizer in a 100 µL of lysis buffer containing 5% sodium deoxycholate, 100 mmol/L Tris-HCl (pH 8.5), 1 mg/mL chloroacetamide, and 1.5 mg/mL tris(2-carboxyethyl)phosphine. The lysates were subsequently heated at 95 °C for 15 min. Protein capture and digestion from bone and bone marrow lysates were performed using an automated KingFisher Flex system (Thermo Fisher Scientific).^119^ Briefly, proteins were captured using MagReSyn HILIC magnetic microspheres (ReSyn Biosciences, Pretoria, South Africa). Protein-bound beads were then digested with 0.5 µg of MS-grade trypsin (Thermo Fisher Scientific) in 50 mmol/L triethylammonium bicarbonate buffer (Sigma-Aldrich) at 37 °C. The tryptic peptides were sequentially washed with 95% (v/v) acetonitrile (ACN) and 70% ethanol (EtOH). Digestion was terminated by acidification with 2% formic acid. Peptides were subsequently desalted using a C18-based desalting procedure, eluted with 0.1% trifluoroacetic acid (TFA) in 50% ACN, and dried by speed vacuum. Thereafter, dried peptides were reconstituted in 0.1% TFA for subsequent mass spectrometry analysis.

One microgram of desalted peptides was loaded onto 25 cm Aurora columns (IonOptiks, Australia) using an RSLC nano µHPLC system coupled to a Fusion Lumos mass spectrometer. Peptide separation was achieved with a 70-min linear gradient ranging from 5% to 30% acetonitrile in 0.5% acetic acid. The mass spectrometer was operated in data-independent acquisition (DIA) mode, collecting MS scans from 350–1 650 Da at 120k resolution, followed by MS/MS acquisition across 45 windows with 0.5 Da overlap (200–2 000 Da range) at 30k resolution and a normalized collision energy (NCE) of 28. The raw data were processed using DIA-NN 2.0 software with spectral matching performed against the Mus musculus UniProt protein database.^120^ Normalization was conducted based on the total peptide abundance across LC-MS runs. GSEA was done using GSEA software (version 4.4.3, Broad Institute, USA).

### Statistical analysis

Data are presented as mean ± SEM or as violin plots, as indicated in the figure legends, with individual data points representing each biological replicate. The solid line represents the median and the dashed line denotes the interquartile range. Statistical analysis was performed using a two-way analysis of variance (ANOVA) to determine the effect of the rbsAb treatment and CKD status on bone and BMAT alterations. Correlations between individual parameters were performed using Spearman correlation. As the plasma DKK1 levels were not normally distributed, the data were analysed using the Mann–Whitney test with Bonferroni correction for multiple comparisons. Statistical analysis was implemented using GraphPad Prism software (GraphPad Software, Inc., USA) and R studio (for cortical bone and BMAT analysis), and statistical significance was shown as; *P < 0.05; ** *P* < 0.01 and *** *P* < 0.001. For differential expression of proteomic profile, the data were processed using Perseus software.^121^ Log₂ transformation was applied to the data, and missing values were imputed based on a normal distribution. Pairwise comparisons between groups were conducted using a two-sample *t*-test. Proteins exhibiting a *P*-value of less than 0.05 and a fold-change greater than 1.5 were considered significantly altered between the compared groups.

## Supplementary information

Suppl Figure 1. Correlation analysis

Suppl Figure 2. Proteomic profiling of cortical bone and bone marrow

Suppl Table 1

Reviewer access details for Proteomics data

## Data Availability

The proteomics data including raw files and search results have been deposited to the ProteomeXchange Consortium via the PRIDE partner repository with the dataset identifier PXD069117.
