# Supplementary material for: Bispecific antibody against sclerostin and DKK1 improves bone health and reduces bone marrow adipose tissue accumulation in experimental chronic kidney disease
Source: Bone Res. 2026 Jul 15;14:73. doi: 10.1038/s41413-026-00556-y (PMC13373193; doi:10.1038/s41413-026-00556-y)
Supplement: Supplementary file 1 — Suppl Figure 1. Correlation analysis [file 41413_2026_556_MOESM1_ESM.pdf]

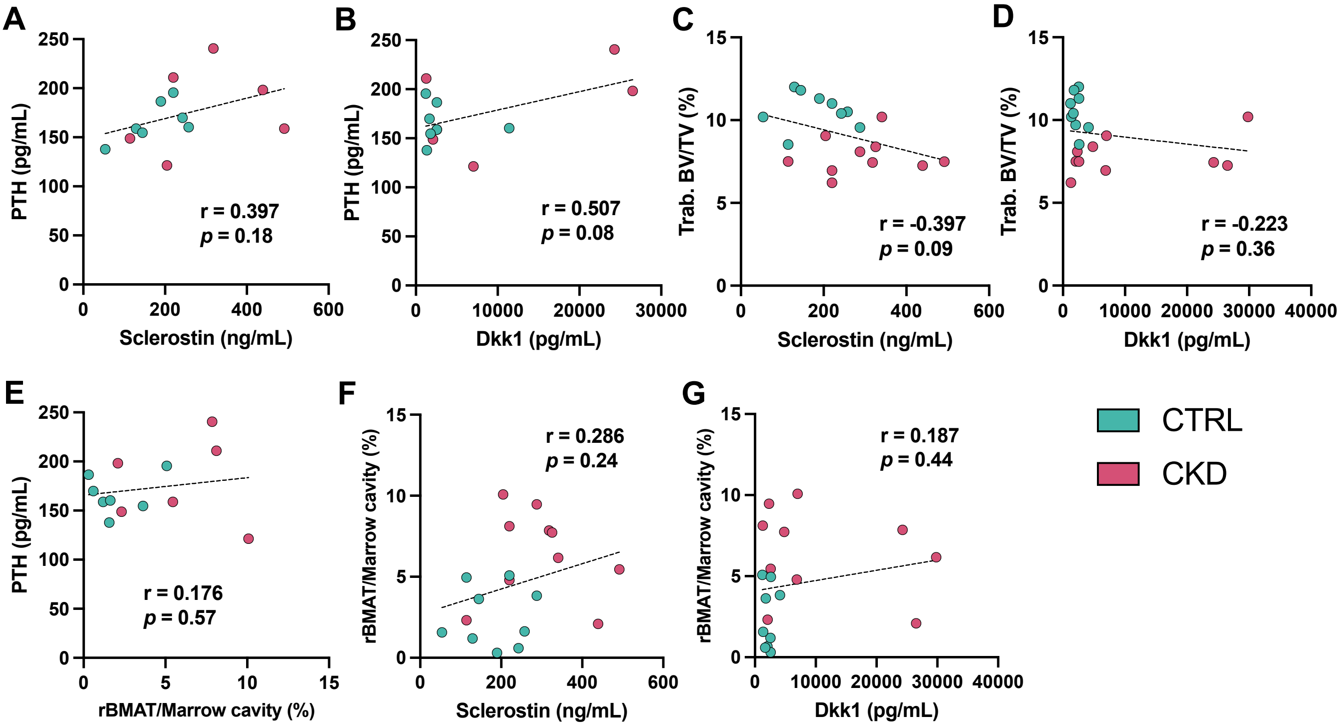

Suppl Figure 1. Correlation analysis. Plasma sclerostin and DKK1 levels of vehicle treated CTRL and CKD mice were not correlated to (A, B) plasma PTH; (C, D) trabecular BV/TV and (F, G) rBMAT. No correlation was found between (E) rBMAT and plasma PTH in vehicle treated CTRL and CKD mice. Correlations between individual parameters were performed using Pearson correlation.
