# Supplementary material for: Bispecific antibody against sclerostin and DKK1 improves bone health and reduces bone marrow adipose tissue accumulation in experimental chronic kidney disease
Source: Bone Res. 2026 Jul 15;14:73. doi: 10.1038/s41413-026-00556-y (PMC13373193; doi:10.1038/s41413-026-00556-y)
Supplement: Supplementary file 2 — Suppl Figure 2. Proteomic profiling of cortical bone and bone marrow [file 41413_2026_556_MOESM2_ESM.pdf]

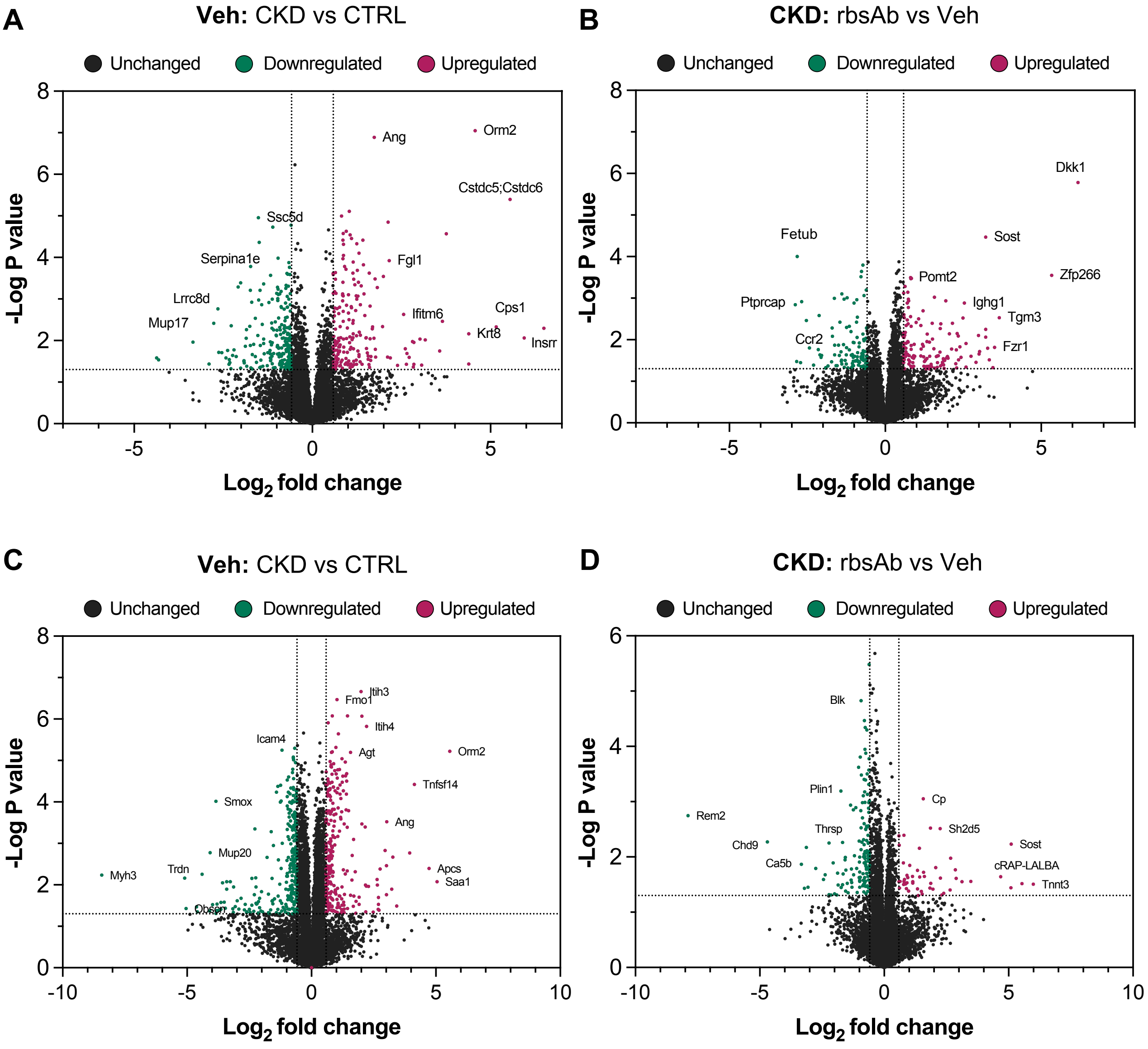

Suppl Figure 2. Proteomic profiling of cortical bone and bone marrow. Volcano plots illustrate global differential protein expression in the tibia (A, B) and the bone marrow (C, D) of CKD mice and mice treated with antibody.
