# Supplementary material for: Bispecific antibody against sclerostin and DKK1 improves bone health and reduces bone marrow adipose tissue accumulation in experimental chronic kidney disease
Source: Bone Res. 2026 Jul 15;14:73. doi: 10.1038/s41413-026-00556-y (PMC13373193; doi:10.1038/s41413-026-00556-y)
Supplement: Supplementary file 3 — Suppl Table 1 [file 41413_2026_556_MOESM3_ESM.pdf]

Supplementary Table 1 Primers for qPCR

Gene	Forward (5`-3`)	Reverse (5`-3`)
Adipoq	AAGAAGGACAAGGCCGTTCTCTT	GCTATGGGTAGTTGCAGTCAGTT
Cebpa	TGGACAAGAACAGCAACGAG	TCACTGGTCAACTCCAGCAC
Fabp4	TGGAAGCTTGTCTCCAGTGA	AATCCCCATTTACGCTGATG
Lipe	CCTTGTGGCTTGCGCTCTGGA	CCGCGCGAACATGACCGAGT
Pparg2	CCAGAGCATGGTGCCTTCGC	TTCCGAAGTTGGTGGGCCAGA
Pnpla2	CGGCTTCCTCGGGGTCTAC	CGCGCTCATGGCAATCAG
Runx2	ACCATAACAGTCTTCACAAATCCT	CAGGCGATCAGAGAACAAACTA
Sp7	ATGGCGTCCTCTCTGCTTG	TGAAAGGTCAGCGTATGGCTT
Col1a1	ACATGTTCAGCTTTGTGGACC	TAGGCCATTGTGTATGCAGC
Alpl	GGGACGAATCTCAGGGTACA	AGTAACTGGGGTCTCTCTC
Mepe	AGAAATATCACGCAGCCTGTAA	GGAGACTTAGCATCATTGACATC
Bglap	CCGGGAGCAGTGTGAGCTTA	TAGATGCGTTTGTAGGCGGTC
Tnfrsf11b	CCTTGCCCTGACCACTCTTA	CACACACTCGGTTGTGGGT
Tnfsf11	CAGCATCGCTCTGTTCCTGTA	CTGCGTTTTCATGGAGTCTCA
sost	TGAGAACAACCAGACCATGAAC	TCAGGAAGCGGGTGTAGTG
Dkk1	CCGGGAACTACTGCAAAAAT	CCAAGGTTTTCAATGATGCTT
Ctsk	GAAGAAGACTCACCAGAAGCAG	TCCAGGTTATGGGCAGAGATT
Mmp9	CAAAGACCTGAAAACCTCCAAC	GACTGCTTCTCTCCCATCATC
Acp5	AGCAGCCAAGGAGGACTACG	TGGCTAACAATGGTCGCAAG
Nfatc1	GGCTGGTCTTCCGAGTTCACATC	GCTGTCTGTGCTCTGCTTCTCC
Ppia	CACCGTGTTCTTCGACATCA	CAGTGCTCAGAGCTCGAAAGT
