# Supplementary material for: Bispecific antibody against sclerostin and DKK1 improves bone health and reduces bone marrow adipose tissue accumulation in experimental chronic kidney disease
Source: Bone Res. 2026 Jul 15;14:73. doi: 10.1038/s41413-026-00556-y (PMC13373193; doi:10.1038/s41413-026-00556-y)
Supplement: Supplementary file 4 — Reviewer access details for Proteomics data [file 41413_2026_556_MOESM4_ESM.docx]

**Reviewer access details for Proteomics data**

Log in to the PRIDE website using the following details:

**Project accession:** PXD069117

**Token:** JSYnsIgYIATX

Alternatively, reviewer can access the dataset by logging in to the PRIDE website using the following account details:

**Username:** [reviewer_pxd069117@ebi.ac.uk](mailto:reviewer_pxd069117@ebi.ac.uk)

**Password:** DtM6jshO94Z4
